# Construction and Application of a Plasmid-Based Signal Peptide Library for Improved Secretion of Recombinant Proteins with *Priestia megaterium*

**DOI:** 10.3390/microorganisms10040777

**Published:** 2022-04-05

**Authors:** Janine Mayer, Tobias Knuuti, Lisa Baumgarten, Elise Menke, Lena Bischoff, Boyke Bunk, Rebekka Biedendieck

**Affiliations:** 1 Institute of Microbiology and Braunschweig Integrated Centre of Systems Biology (BRICS), Technische Universität Braunschweig, Rebenring 56, 38106 Braunschweig, Germany; janine.mayer@tu-braunschweig.de (J.M.); tobiasknuuti@gmx.de (T.K.); lisa.baumgarten@t-online.de (L.B.); e.menke@tu-braunschweig.de (E.M.); l.bischoff@tu-braunschweig.de (L.B.); 2 Leibniz Institute DSMZ-German Collection of Microorganisms and Cell Cultures, Inhoffenstraße 7, 38124 Braunschweig, Germany; boyke.bunk@dsmz.de

**Keywords:** *Priestia megaterium*, genetic tools, signal peptide (SP) library, protein secretion, recombinant proteins, penicillin G acylase

## Abstract

The secretion of recombinant proteins plays an important role in their economic production and purification. The secretion efficiency depends on the responsible signal peptide (SP) in combination with the target protein and the given host and cannot be predicted so far. Due to its high plasmid stability, the lack of alkaline extracellular proteases and only few contaminating extracellular host proteins, *Priestia megaterium* provides a promising alternative to common *Bacillus* species. For the development of an easy and fast cloning and screening system to identify the SP best suited to a distinct protein, a plasmid-based SP library containing all predicted 182 Sec-dependent SPs from *P. megaterium* was established. The splitting of the SPs into 10 groups of individual multi-SP plasmids (pMSPs) allows their grouped amplification and application in screening approaches. The functionality of the whole library was demonstrated by enhancing the amount of the already well-secreted α-amylase AmyE by 1.6-fold. The secretion of a novel penicillin G acylase, which remained as insoluble protein inside the cells, as its native SP is unsuitable for secretion in *P. megaterium*, could be enhanced even up to 29-fold. Overall, only around 170 recombinant *P. megaterium* clones based on 50 inserted SPs had to be screened to achieve sufficient amounts for further enzyme characterizations. Thus, this newly developed plasmid-based genetic tool applicable for *P. megaterium* and also other *Bacillus* species facilitates the identification of suitable SPs for secretion of recombinant proteins.

## 1. Introduction

For decades, the biotechnological production of recombinant proteins has played an important role in industry [1]. The emergence of recombinant DNA technology in the 1970s led to an enormous increase of potential applications in the area of biopharmaceuticals and industrial enzymes [2,3]. The secretion of recombinant proteins to the extracellular environment has advantages over their intracellular production. The downstream processing is easier, non-denaturing, and cost-effective due to the purification of correctly folded proteins from the cell-free culture broth without cell disruption [4,5]. Furthermore, the secretion of recombinant proteins can facilitate continuous cultivation [6]. Due to the architecture of their cell wall, Gram-positive bacteria are especially well suited to the secretion of proteins. They are characterized by an intrinsic capacity to secrete proteins directly into the surrounding medium because of the lack of an outer membrane [4,7].

There are two main pathways for protein transport across the plasma membrane in bacteria: the general secretion (Sec-dependent) pathway and the twin-arginine translocation (Tat) pathway [7]. On the Sec-dependent pathway, proteins are transported unfolded across the plasma membrane and folded in the periplasm or the extracellular environment, whereas proteins which were already folded in the cytoplasm are exported via the Tat pathway [5]. In bacteria, the ubiquitous Sec-dependent pathway is the major secretion mechanism for proteins exported from the cytosol [8]. Studies in Gram-positive *Bacillus subtilis* have shown that more than 90% of all secreted proteins are transported via this pathway in this organism guided by so-called signal peptides (SPs) [9]. SPs are responsible for protein translocation by enabling the recognition and the transport of corresponding proteins to the membrane. After the transport across the plasma membrane, SPs are cleaved by signal peptidases (SPases) [10]. Sec-dependent SPs are localized N-terminally of the protein to be secreted. They consist of a positively charged amino-terminal n-region, which is thought to interact with the translocation machinery, the central hydrophobic h-region, which can form hairpin-like structures, and the polar carboxy-terminal c-region, which carries the SPase recognition sequence so that the SP can be cleaved from the translocated protein. All three regions contribute to efficient protein export [5,11,12,13].

Naturally, each protein to be exported has its own specific SP. For the secretion of heterologous proteins with a specific host, a suitable SP is needed. The probability of an N-terminal amino acid sequence to be a SP in bacteria or eukaryotes can be predicted bioinformatically using diverse web tools such as PrediSi [14] or SignalP [15,16]. This way, 173 natural Sec-dependent SPs were predicted for the Gram-positive model organism *B. subtilis* [17]. Nevertheless, the transport efficiency depends on the combination of the SP with the particular protein and cannot be predicted. Hence, a large number of SPs must be screened experimentally for each recombinant protein to achieve the best secretion efficiency in the given host [5]. For *B. subtilis*, all predicted Sec-dependent SPs were amplified individually by PCR and tested for their efficiency on the secretion of cutinase and esterase [17], α-amylase [18,19], and a protease [20] resulting in drastically different amounts of secreted protein depending on the used SP. A SP which was most effective for one protein could be really weak for another [17,21]. This system is available commercially, so that the 173 Sec-dependent SPs can be used directly as a mix of PCR-products followed by a high-throughput screening system for each individual target protein [18].

Apart from the well-established Gram-positive secretor *B. subtilis*, *Priestia megaterium* (formerly *Bacillus megaterium*) was developed for recombinant protein production and secretion during the past 30 years [4,22]. *P. megaterium* offers various advantages for recombinant protein production. Like all Gram-positive bacteria, *P. megaterium* contains no endotoxins, in contrast to Gram-negative bacteria, and is capable of producing high yields of recombinant protein metabolizing inexpensive substrates [23]. *P. megaterium* exhibits a high stability of recombinant plasmids even in the absence of antibiotics [24,25] and does not own extracellular alkaline proteases [26]. For further improvement of the stability of the secreted protein, the gene encoding the major extracellular protease NprM of *P. megaterium* DSM 319 was inactivated, resulting in the strain MS941 [27]. Existing plasmid systems based on a strong optimized xylose-inducible promoter system allow secretion of recombinant proteins in excess, representing more than 70% of all secreted proteins, enabling an easy purification directly from the cell-free medium [28,29,30].

To further expand the range of applications of *P. megaterium* to make even better use of the advantages mentioned above [22,23,31], the aim of this study was to establish a simple and rapid cloning and screening system allowing the identification of SPs best suited to efficient secretion of individual recombinant proteins. For this, a plasmid-based SP library including all Sec-dependent SPs from *P. megaterium* was designed. To validate the functionality of this system, the model enzyme α-amylase AmyE from *B. subtilis* was used. Further, the new tool was applied to find a suited SP for a novel penicillin G acylase from *Bacillus massiliogorillae* that is hardly secreted with its native SP in *P. megaterium* [30]. An upscaling from microtiter plate to shake flask scale could verify the functionality of the established plasmid-based SP test system.

## 2. Materials and Methods

### 2.1. Bacterial Strains and Construction of Expression Vectors

The cloning was carried out in *Escherichia coli* strain DH10B (Life Technologies, Carlsbad, USA). For screening experiments and protein production, the protease-deficient *P. megaterium* strain MS941 was used [27].

The plasmid p3STOP1623hp [28] (Table S1) served as the origin for the construction of expression vectors. In one cloning step, its multiple cloning site (MCS) was extended (restriction sites for KasI, MluI, AvrII, NotI, NheI, and AgeI) and the coding sequence of a His_6_-tag was integrated via oligos (Table S2) using the restriction site AgeI, resulting in plasmid pTKSP0. Next, the *amyE* gene of *B. subtilis* 168 (GenBank: CAB12098.2) encoding the α-amylase AmyE was amplified by PCR from genomic DNA without its native signal peptide (SP) and integrated in pTKSP0 via the restriction sites NgoMIV and AgeI, resulting in the plasmid pTKSP*amyE*0. As a control, the coding sequence of the previously used SP of lipase A (*sp_lipA_*, [32]) from *P. megaterium* was amplified via PCR primer (Table S2) from pEJBmD1.3scFv [33] and inserted in pTKSP*amyE*0 via the restriction sites BsrGI and NgoMIV yielding pTKSP*amyE*lipA. Furthermore, the *bmaspga* gene was amplified by PCR from vector pRBBm317 [30] without its SP coding sequence and integrated in pTKSP0 via the restriction sites NgoMIV and NheI, resulting in the plasmid pJMBm75. The success of cloning procedures was verified by DNA sequencing (Microsynth Seqlab GmbH, Göttingen, Germany).

### 2.2. Construction of Multisignal Peptide Plasmids (pMSP)

Sec-dependent SPs of *P. megaterium* DSM 319 were identified with the online tool SignalP 4.0 [15]. The SP coding sequences were sorted by size and divided on ten multisignal peptide plasmids (pMSPs, Tables S3–S12). The coding sequences of all SPs in groups of 18 and 20 SPs, respectively, were arranged in alternating orientations and separated by the restriction sites BsrGI (5′) and NgoMIV (3′). All pMSPs were synthesized by General Biosystems (Morrisville, NC, USA).

### 2.3. Cloning of SP Coding Sequences into Expression Vectors

For cloning of SP coding sequences from pMSPs into the obtained plasmids, the multi-SP plasmids (pMSP1–10) and the expression vector pTKSP*amy*E0 and pJMBm75, respectively, were digested with BsrGI and NgoMIV. To prevent ligation of more than one SP coding sequence in one vector, the ends of the obtained SP coding sequences were dephosphorylated with shrimp alkaline phosphatase (rSAP). Subsequently, the DNA fragments were separated by agarose gel electrophoresis and the corresponding band for the expression vector and SP mix were excised and purified. While the digested vectors were purified using the NucleoSpin PCR and Gel Clean-Up Kit (Macherey-Nagel, Düren, Germany), the SP mix was purified using the QIAEX II Gel Extr action Kit (QIAGEN, Hilden, Germany). Four µL (50 ng) of purified vector fragment and 4.5 µL (200 ng) of SP mix were ligated using T4 DNA ligase. Next, *E. coli* DH10B cells were transformed with ligation preparations to generate a higher number of isolated colonies representing clones with varying SPs. All clones were suspended together in LB medium (10 g L^−1^ tryptone, 5 g L^−1^ yeast extract, 5 g L^−1^ NaCl) and the plasmid mix was purified in one step using the NucleoSpin Plasmid Kit (Macherey-Nagel, Düren, Germany). Finally, *P. megaterium* MS941 was transformed with the plasmid mix by protoplast transformation [34]. Isolated single colonies were applied for the screening approach.

### 2.4. Cultivation in a Microbioreactor System

For the screening of different SPs, the cultivation of *P. megaterium* MS941 containing plasmids with differing SPs was carried out in 48-well flower plates in a BioLector system (m2p-labs, Baesweiler, Germany) at 37 °C and 1400 rpm for 24 h. One mL of LB medium supplemented with 10 mg L^−1^ tetracycline, 0.5% (*w*/*v*) xylose and, for PGA secretion, 2.5 mM of CaCl_2_ was used per well. Each well was inoculated with corresponding overnight pre-cultures, resulting in an initial optical density of 578 nm (OD_578_) of 0.055. During cultivation, the growth was monitored by scattered light at 620 nm. After 24 h, the flower plate was centrifuged for cell-harvesting (3300× *g*, 20 min, 4 °C) and the cell-free supernatant was used for activity determination.

### 2.5. Cultivation in Shake Flasks and Protein Analyses

For shake flask cultivations, overnight pre-cultures of corresponding *P. megaterium* MS941 plasmid strains were used to inoculate 50 mL LB medium supplemented with 10 mg L^−1^ tetracycline and 2.5 mM CaCl_2_. The cultures were incubated at 37 °C and 200 rpm. At an OD_578_ of 0.3 to 0.5, the induction of recombinant gene expression was performed by adding 0.5% (*w*/*v*) xylose. After further cultivation at 37 °C and 200 rpm for 22 h, cells were separated from the protein-containing supernatant by centrifugation.

For the analysis of extracellular proteins, these were precipitated from the cell-free supernatant by addition of ammonium sulfate. For this, 1.5 mL of cell-free supernatant was shaken with 660 mg ammonium sulfate for 2 h at 4 °C with shaking at 1000 rpm. After centrifugation at 17,000× *g* and 4 °C for 30 min, the precipitated proteins were suspended in 20 μL ddH_2_O and analyzed by SDS-PAGE.

For the analysis of intracellular proteins, cell sediments of two OD equivalents were suspended in 20 μL digestion buffer (100 mM Na_3_PO_4_, 5 mg mL^−1^ lysozyme, 2 μL mL^−1^ benzonase, pH 6.5) and incubated for 30 min at 37 °C and 1000 rpm. After centrifugation (17,000× *g*, 4 °C, 15 min), the supernatant was used to analyze soluble intracellular proteins by SDS-PAGE. The sediment obtained after centrifugation was suspended in 20 μL 8 M urea. After centrifugation (17,000× *g*, 4 °C, 10 min), the supernatant was used to analyze the insoluble intracellular proteins by SDS-PAGE.

### 2.6. Amylase Activity Assay

The Phadebas^®^ Amylase test (Kristianstad, Sweden) was used to determine the enzyme activity of α-amylase in the cell-free supernatant. By degradation of starch by α-amylase the embedded dye is released. The measured absorption at 620 nm is proportional to the converted substrate and thus to the amount of secreted α-amylase. One hundred microliters of cell-free culture medium obtained after centrifugation were mixed with 800 µL Phadebas^®^ Amylase test solution and incubated at 37 °C with shaking of 1400 rpm for 15 min. Subsequently, the reaction was stopped by the addition of 600 µL of 500 mM NaOH. The mixture was centrifuged (21,000× *g*, 10 min) and the supernatant was analyzed at 620 nm.

### 2.7. PGA Activity Assay

For PGA activity screening, the alternative substrate 2-nitro-5-[(phenylacetyl) amino]-benzoic acid (NIPAB) was used [35]. To each well of a 96-well-plate with flat bottom (Kisker Biotech, Steinfurt, Germany) 90 µL of substrate solution (600 mg L^−1^ NIPAB, 9.41 mM NaH_2_PO_4_, 40.59 mM Na_2_HPO_4_, pH 7.5) was added. To start each reaction, 10 µL of cell-free supernatant was added and mixed. The plate was incubated at 37 °C and the absorption (A) at 405 nm (extinction coefficient ε of 8.98 cm^2^ µmol^−1^) was measured every six seconds for three minutes using a microplate reader (Tecan, Männedorf, Switzerland). The enzymatic activity EA [U mL^−1^] was calculated using the following formula with the reaction volume V_R_ [cm^3^], the sample volume V_E_ [cm^3^], and the layer thickness d [mm].

(1) $$\mathrm{EA}=\frac{\frac{\mathrm{dA}}{\mathrm{dt}}\cdot V_{R}}{\varepsilon \cdot d\cdot V_{E}}$$

### 2.8. Colony PCR for Identification of Signal Peptides

For the identification of the corresponding SPs, colony PCR was performed and the resulting fragments were sequenced. *P. megaterium* colonies were suspended in lysis buffer containing 150 mM Tris-HCl (pH 8), 1 mg mL^−1^ lysozyme, and 100 µg mL^−1^ proteinase K and incubated for 20 min at 37 °C and 400 rpm, followed for another 20 min at 55 °C and 400 rpm. Subsequently, the enzymes were heat inactivated at 95 °C and the samples centrifuged at 21,000× *g* for 10 min. The supernatant was used as template for the PCR reaction. For colony PCR, taq polymerase and primers SeqpXylA_fw and SeqpMM1520_rv were used (Table S2). The resulting fragments were purified and the coding sequences of the SPs were identified by DNA sequence analysis. Alternatively, *P. megaterium* colonies were directly applied for sequencing using Ecoli NightSeq (Microsynth Seqlab GmbH, Göttingen, Germany), which was proven to work for Gram-positive *P. megaterium* before.

## 3. Results

Secretion of recombinant proteins, in contrast to intracellular accumulation of proteins, offers advantages such as simplified product recovery or the possibility of continuous cultivation [34]. For this purpose, the Gram-positive soil bacterium *P. megaterium* is of biotechnological relevance as it secretes recombinant proteins directly into the surrounding medium with only low amounts of host proteins compared to other secreting bacteria such as *B. subtilis* [30,36].

The amount of a secreted protein depends on the combination of the N-terminal signal peptide (SP), responsible for translocation, and the protein itself. However, as bioinformatics predictions of efficiency are not yet possible, for each protein of interest the most effective SP has to be found experimentally [5].

### 3.1. Construction of a Signal Peptide Test System in P. megaterium

**Figure 1:**
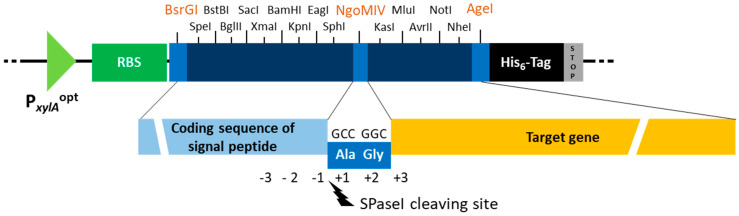
Schematic representation of the molecular components of the plasmid-based SP test system in *P. megaterium* based on the plasmid pTKSP0. The SP coding sequences including their appropriate native start codon can be cloned in the 5′ part of the MCS, ideally using the BsrGI (5′) and NgoMIV (3′) sites. The target gene can be cloned in the 3′ part of the MCS using the corresponding recognition sites optional as fusion to the His_6_-tag coding sequence. On the protein level, the inserted NgoMIV restriction site leads to the indicated additional alanine and glycine residues between SP and target protein, which now represent the +1 and +2 positions relative to the signal peptidase I (SPaseI) cleaving site. The SPaseI cleaving site is defined as position −3, −2, −1 (last residues of the SP) and position +1 of the target protein. P*_xylA_*^opt^—optimized xylose inducible promoter; RBS—ribosome binding site.

First, we developed a plasmid setup that allows a simple and standardized exchange of both the coding sequence of the SP and the corresponding gene of the protein to be secreted. Thereby, optimal combinations of SP and protein can be identified, while the plasmid scaffold remains unchanged. Therefore, the secretion efficiency is only affected by the SP and in addition the amount of secreted protein should provide a direct statement about the SP functionality and secretion efficiency. The new plasmid system is based on the plasmid p3STOP1623hp, an established shuttle vector for cloning in *E. coli* and xylose-dependent recombinant protein production in *P. megaterium* [28]. Downstream of the optimized xylose inducible promoter P*_xylA_*^opt^, a multiple cloning site (MCS) is located [37], which was extended by five recognition sites near the 3′ end in this study. In addition, the coding sequence of a His_6_-tag was introduced downstream of the MCS followed by a stop codon to allow an affinity chromatographic purification of the target protein, resulting in the new plasmid pTKSP0 (Figure 1). Due to the large selection of restriction sites, the construction of pTKSP0 allows the easy cloning of the SP coding sequence in the 5′ part of the MCS and of the target gene in the 3′ end.

For cloning of the SP coding sequence at the 5′ end of the MCS, restriction enzyme BsrGI can be chosen, whose recognition site is located between the ribosome binding site (RBS) and the corresponding start codon of the following coding sequence, so that the original 5′ end remains intact, resulting in the native N-terminus of the SP. The fusion of the SP coding sequence and the target gene results in the insertion of two amino acids on protein level between SP and target protein (Figure 1). These two residues at the so-called +1 and +2 position relative to the signal peptidase I (SPaseI) restriction site can affect the effectivity of SPase I. It depends on the N-terminal amino acid of the protein (+1) and on the C-terminal residues of the SP itself at positions −3, −2, −1 [10], while the latter are unchanged during the cloning procedure. Furthermore, these two additional +1 and +2 amino acids may influence the properties of the protein, since they remain at the N-terminus of the secreted protein after cleavage of the SP. Hence, the restriction enzyme NgoMIV was chosen, whose in-frame recognition sequence gccggc results in the insertion of the small and uncharged alanine and glycine residues with expectation of the least effect due to steric hindrance or other interactions on the secreted protein. In addition, both amino acids have been described as not negatively influencing the cleavage of the SP by SPaseI [10,38]. At the 3′ end, the in-frame recognition site of AgeI results in the least amino acid residues between protein of interest and His_6_-tag but all other cleavage sites are also possible, especially for cloning without His_6_-tag fusion.

### 3.2. Identification of Sec-Dependent Signal Peptides from P. megaterium DSM 319

To identify Sec-dependent SPs used for protein secretion with *P. megaterium*, all annotated open reading frames (ORFs) of *P. megaterium* strain DSM 319 were translated to protein sequences. Possible N-terminal located Sec-dependent SPs were predicted using the web-based program SignalP 4.0 [15], based on a calculated discrimination score (d score), a measure to distinguish SPs from non-SPs. Amino acid sequences with a d score greater than 0.45 were considered as Sec-dependent SPs for *P. megaterium*. According to this procedure, 182 SPs were identified in the *P. megaterium* DSM 319 genome with lengths between ten and 56 amino acids (Tables S3–S12).

### 3.3. Construction of Multisignal Peptide Plasmids (pMSPs) for P. megaterium

**Figure 2:**
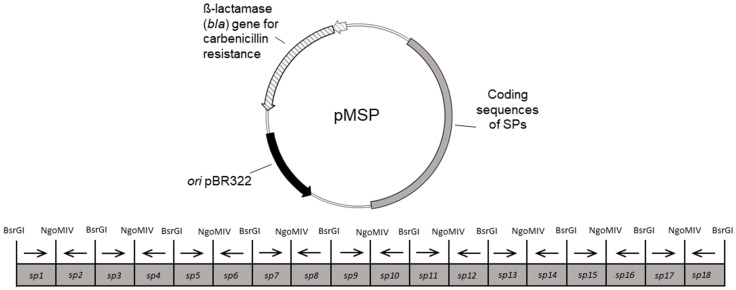
Map of a multi-signal peptide plasmid (pMSP). All pMSPs carry a resistance gene (*bla*) for carbenicillin resistance (striped), the *ori* of pBR322 for *E. coli* (black) and a DNA cassette encoding the individual SPs (gray). This DNA cassette is shown in more detail in the image below. It contains coding sequences of 18–20 SPs from *P. megaterium* DSM 319 flanked by BsrGI (upstream) and NgoMIV (downstream) recognition sites. The black arrows indicate the respective orientation of coding sequences of the SPs.

In previous publications, the coding sequences of the SPs were often individually amplified by PCR [17,19], which allows for individual mixing of different SPs but also implies a higher workload and higher cost. Here we present a new approach in which the coding sequences of the SPs were located on plasmids separated by restriction sites so that their fast and easy amplification, restriction, and cloning is possible. For this, the coding sequences of the 182 predicted SPs of *P. megaterium* were arranged on ten multi-signal peptide plasmids (pMSPs) (Figure 2). pMSP1 to pMSP9 each contain 18 and pMSP10 contains 20 coding sequences for SPs. The sequences were sorted by size, so that the longest SP sequences were grouped on pMSP1 going up to pMSP10, which is carrying the shortest sequences (Tables S3–S12) to prevent possible preferential incorporation of long or short sequences into the targeting vector. The SP coding sequences were assembled as cassettes on the plasmids, arranged in alternating orientation so that BsrGI is at the 5′ and NgoMIV at the 3´ end for ligation in correct orientation into the target vector and synthesized by General Biosystems (Morrisville, NC, USA) (Figure 1 and Figure 2).

**Figure 3:**
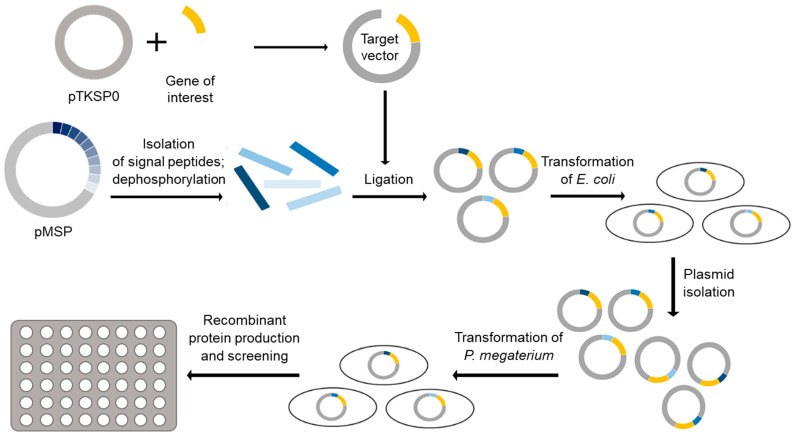
Illustration of cloning and screening procedure to identify efficient combinations of SP and target protein that lead to high secretion in recombinant *P. megaterium*. Coding sequences of SPs are shown in blue, the target gene in yellow.

To investigate the functionality of the developed plasmid-based SP test system, a screening system in *P. megaterium* was established (Figure 3). First, the target gene has to be cloned into pTKSP0 using NgoMIV as 5′ recognition site and one of the following 3′ sites of the MCS (Figure 1). Subsequently, both the constructed target vector and one or more pMSPs have to be digested using BsrGI and NgoMIV. The fragments representing the SP coding sequences need to be dephosphorylated to prevent incorporation of multiple sequences in one target vector. After ligation of the SP mix with the target vector, *E. coli* has to be transformed with the corresponding ligation reaction, which allows a rapid amplification of the plasmid mix. A direct transformation of *P. megaterium* is not possible due to its low transformation efficiency [39]. After isolation of the amplified plasmid mix from all *E. coli* clones, *P. megaterium* can be transformed with the mixture and individual clones screened according to their secretion capability. An individual activity assay has to be established for each applied protein in order to use activity as a measure of the amount of protein secreted.

### 3.4. Screening of Signal Peptides of All pMSPs Regarding Secretion of Model Protein α-Amylase

To validate the new system, the α-amylase AmyE of *B. subtilis* was chosen as a model protein to observe, compare, and evaluate the secretion success achieved by different SPs. AmyE is characterized by its high stability, does not inhibit growth, and has no toxic effects on the host organism [40,41]. Furthermore, the secretion of amylases is widely used as a reporter system as it allows screening in liquid cultures as well as on starch-containing LB agar by detection via a colorimetric assay due to its enzymatic reaction, in which α-1-4-glycosidic bonds of polysaccharides are endohydrolytically cleaved [40].

**Figure 4:**
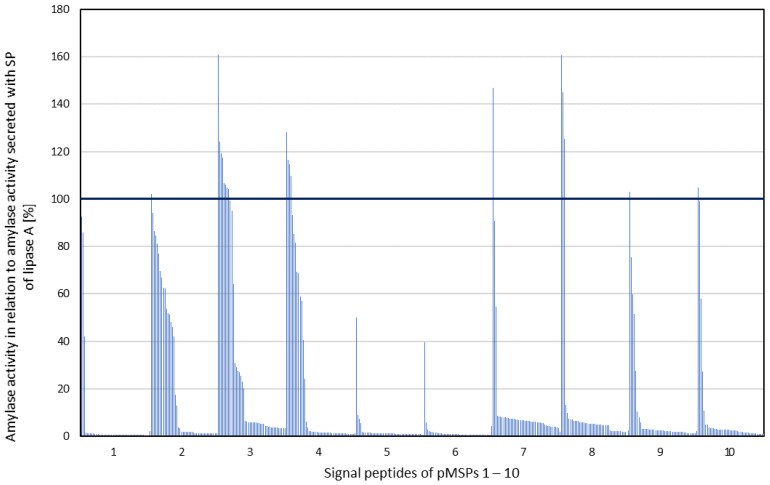
Activity of α-amylase AmyE in the cell-free supernatant recombinantly secreted by *P. megaterium* MS941 with SPs from pMSP1 to 10. The measured enzyme activity was related to the scattered light at 620 nm of the culture. Enzyme activity is given as a percentage and refers to the positive control (100%), in which the α-amylase is recombinantly secreted by the SP from lipase A of *P. megaterium* ATCC 9885. Cultivation was performed in a microbioreactor system with 1 mL of LB medium supplemented with tetracycline (10 μg mL^−1^) and 0.5% (*w*/*v*) xylose at 37 °C and 1400 rpm. After 24 h, α-amylase activity in the cell-free culture supernatant was determined photometrically (OD_620_).

The *amyE* gene and subsequently the SP coding sequences from pMSP1 to 10 were introduced into pTKSP0. After amplification in *E. coli*, *P. megaterium* was transformed with the obtained plasmid mixes, yielding 10 libraries of *P. megaterium* clones containing plasmids with *amyE* and SPs from one pMSP each. After cultivation of 45 individual clones per pMSP in a microbioreactor system, the amylase activities were determined in the cell-free supernatant. They were compared to the activity resulting from recombinant AmyE secretion with the SP of the lipase A from *P. megaterium* ATCC 9885 (pTKSP*amyE*lipA), for which previous work has already demonstrated a high secretion efficiency of recombinant proteins with *P. megaterium* [22]. As a negative control, recombinant AmyE-production without SP (pTKSP*amyE*0) was chosen. The amylase activity in the cell-free supernatant of the 450 cultivations was related to the scattered light at 620 nm of the culture as a measure for the formed biomass (Figure 4).

The photometric screening of the SP-AmyE library with respect to the amount of secreted α-amylase in the culture supernatant revealed strong differences ranging from no (0%) to 161% secreted amylase compared to the control. While around 84% of the clones showed no or less than 10% amylase activity in the supernatant compared to the control, for 18 clones (4%) a higher activity of amylase was detected caused by SPs from pMSP2–4 and pMSP7–10, respectively. DNA sequencing revealed that enhanced AmyE secretion was caused by SPs originating from conserved hypothetical proteins with unknown function (nine of the 18 SPs) and also from proteins with functions reaching from flagellum and peptidoglycan biosynthesis (three) to hydrolases (four) and transferases (two) (Table 1).

The length of SPs leading to increased AmyE secretion varied between 21 and 31 amino acids in our study, while SPs ranging from 10 to 56 amino acids were predicted and tested (Table 1 and Tables S3–S12). However, 75% of the *P. megaterium* SPs are in the range of 21 to 31 amino acids, while only 7% of the SPs are shorter and 18% are longer.

The results of the first screening clearly showed the influence of SPs on protein secretion. With the system established here, several SPs could be identified, which led to a very good secretion efficiency for amylase. Although the secretion efficiency for α-amylase AmyE with the SP of lipase A used as control was already high, a further 1.6-fold increase in secretion efficiency could be achieved using the constructed SP library for *P. megaterium*. In a next step, it was of great interest to apply this new system to other proteins with previously poor secretory properties.

### 3.5. Application of the SP Library for Improved Secretion of Penicillin G Acylase

**Figure 5:**
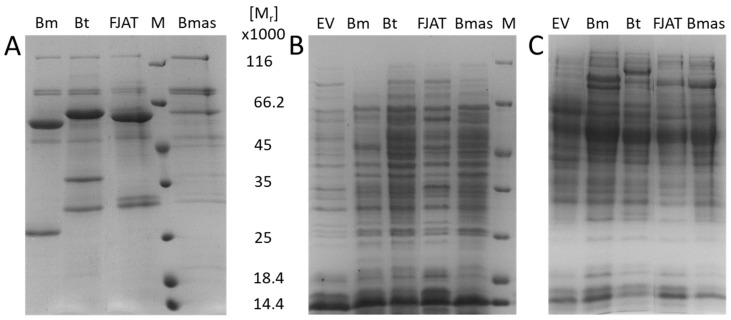
SDS-PAGE analyses of extra- and intracellular proteins during the recombinant production of PGAs with *P. megaterium*. Recombinant *P. megaterium* carrying the plasmids for production of PGAs from *P. megaterium* (pALBm1, Bm), *B. thermotolerans* (pRBBm311, Bt), *B*. sp. FJAT-27231 (pRBBm316, FJAT), and *B. massiliogorillae* (pRBBm317, Bmas), respectively, were cultivated aerobically at 37 °C, recombinant protein production was induced, and samples were taken for the analyses of extra- (**A**, 22 h after induction) and intracellular (**B** + **C**, 3 h after induction) proteins. Intracellular proteins were further separated in soluble (**B**) and insoluble (**C**) fractions. *P. megaterium* carrying an empty vector (EV) served as control. M: protein molecular weight marker.

Next, our SP library was applied to the secretion of industrially relevant penicillin G acylase (PGA), a heterodimeric enzyme with a molecular weight of around 90,000, which is used for the production of semisynthetic β-lactam antibiotics [30,42,43]. Recently, newly identified PGAs from different *Bacillus* species were found to be recombinantly secretable by *P. megaterium* via their Sec-dependent native SPs [30]. However, the amount of recombinant PGA in the cell-free supernatant differed significantly. While the PGAs from *Bacillus thermotolerans* (BtPGA) and *B.* sp. FJAT-27231 (FJAT-PGA) guided by their native SPs were secreted in similar amounts as the industrially used PGA from *P. megaterium* (BmPGA), allowing their purification and characterization, hardly any secretion was observed for the PGA from *B. massiliogorillae* (BmasPGA) even 22 h after induction of the recombinant protein production (Figure 5A) [30].

To exclude ineffective production of BmasPGA in general, the intracellular proteins of plasmid-carrying *P. megaterium* recombinantly producing different PGAs were analyzed. In the patterns of the intracellular soluble proteins, no differences between the strain containing the *pga* encoding plasmid and a negative control could be detected (Figure 5B). When insoluble proteins were analyzed, additional bands representing proteins with a relative molecular weight of 80,000 to 90,000 were observed 3 h after induction of recombinant protein production, which were missing in the negative control. When exported via the Sec-dependent pathway, PGAs are initially produced as a preproprotein with SP, α-subunit, linker, and β-subunit. Only after export, the SP and linker are cleaved and the PGA is folded into its active conformation [44]. Consequently, the observed bands in the patterns of the insoluble proteins could be the not yet or incorrectly folded insoluble preproPGAs. As obviously the export of recombinant BmasPGA with its native SP represents the main bottleneck, it should be improved by screening SPs from *P. megaterium*.

**Figure 6:**
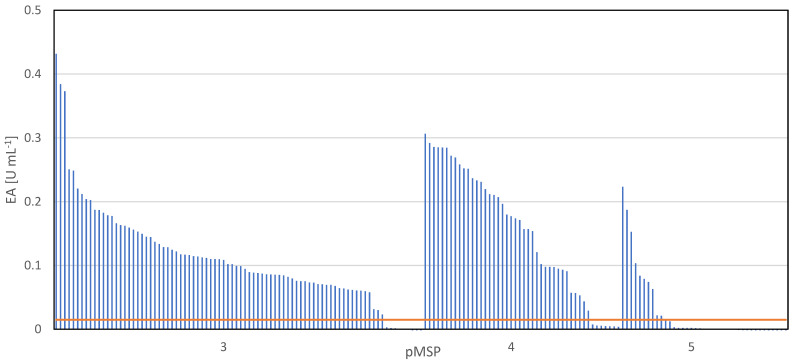
Volumetric enzyme activity (EA) in supernatants from recombinant *P. megaterium* MS941 containing BmasPGA secreted by alternative SPs from pMSP3 to 5. Cultivation was performed in a microbioreactor system at 37 °C and 1400 rpm for 22 h. After centrifugation, enzyme activity was determined in the cell-free supernatant by NIPAB assay at 37 °C. Supernatant of BmasPGA secreted by its native SP served as control (orange line).

After constructing the new target vector (Figure 1) carrying the *pga* gene from *B. massiliogorillae* (pJMBm75), SPs from pMSP3 to 5 were inserted as described above. SPs from pMSP3 and 4 were chosen as they caused the most robust results for the secretion of recombinant AmyE. In contrast, the functionality of SPs from pMSP5 should be tested for BmasPGA as they showed poor secretion for amylase (Figure 4). Single colonies of 171 recombinant *P. megaterium* clones were cultivated in a microbioreactor system for 22 h and the volumetric PGA activity in the cell-free supernatant was determined, serving as an indirect measure of the amount of secreted BmasPGA. By using alternative SPs from *P. megaterium* DSM 319, the secretion of recombinant BmasPGA with *P. megaterium* could be significantly increased (Figure 6).

When cell-free supernatants of 86 clones with SPs from pMSP3 were analyzed, 93% of the clones secreted higher amounts of BmasPGA compared to the secretion with its native SP. Eighty-five percent of the 46 BmasPGA clones with SPs from pMSP4 showed higher PGA activity in the supernatant than the reference strain. In contrast, when SPs from pMSP5 were used, only 28% of the clones resulted in increased activity in the supernatant, while the others resulted in even less secretion of BmasPGA compared to the secretion with the native Bmas-SP (Figure 6). The highest enzyme activities in the supernatant were obtained by SPs from pMSP3 (29-fold increased) followed by pMSP4 (21-fold), while using pMSP5, still 15-fold higher activity compared to the reference strain was observed.

Next, SPs were identified by DNA sequencing. From pMSP3 and 4, SP-coding sequences from all clones were sequenced while for pMSP5 only SP-sequences from clones secreting BmasPGA were investigated. Interestingly, the individual SPs occurred with very different frequencies. For pMSP3, 14 of 18 possible SPs were found in the clones examined. The most frequently found SP was SP 6, which originated from the conserved hypothetical protein BMD_0560 from *P. megaterium* DSM 319, with 38% of all sequenced SPs (Table 2). For clones with SPs from pMSP4, 11 of 18 possible SPs were found but only half as many clones were studied as for pMSP3. The most frequent SP 9 from a protein of the ErfK/YbiS/YcfS/YnhG family occurred in 25% of the clones. In clones with SPs from pMSP5, seven of 18 possible SPs were detected in 12 sequenced clones. The most frequent SP 15 from a conserved hypothetical protein from *P. megaterium* DSM 319 occurred four times. In eight clones across all pMSPs, no SP could be found. This correlates also with nearly no measured activity. In six clones, multiple SPs were detected (Table 2).

In contrast to these results, it was assumed that by amplifying the entire plasmid, all corresponding genes of the SPs would be equally abundant and accordingly equally distributed among the vectors with *bmaspga* gene. However, this was not the case. It would be possible that secondary structures of some SP genes were formed due to complementary segments, which led to the altered insertion rate. Furthermore, several SP genes might align with each other or were attached to the vector by complementary stretches, so that fewer of these SP genes were freely present and less likely to ligate into the target vector.

The highest amount of secreted BmasPGA was achieved by the SP of a β-amylase from *P. megaterium* DSM 319 (SP 2 on pMSP3) with an activity of 0.37 to 0.43 U mL^−1^ and the SP of an extracellular ribonuclease (SP 5 of pMSP3, 0.25 U mL^−1^). In addition, the SPs from a D-alanyl-D-alanine carboxypeptidase (SP 3 of pMSP3), an alkaline phosphatase (SP 7 of pMSP3), an ErfK/YbiS/YcfS/YnhG family protein (SP 9 of pMSP4), two conserved hypothetical proteins, BMD_0470 (ADF37360. 1, SP 11 of pMSP4) and BMD_3362 (SP 2 of pMSP5), a hypothetical protein BMD_2542 (SP 1 of pMSP5), and l-asparaginase II (SP 9 of pMSP5) led to a high amount of secreted BmasPGA of up to 0.43 U mL^−1^.

**Figure 7:**
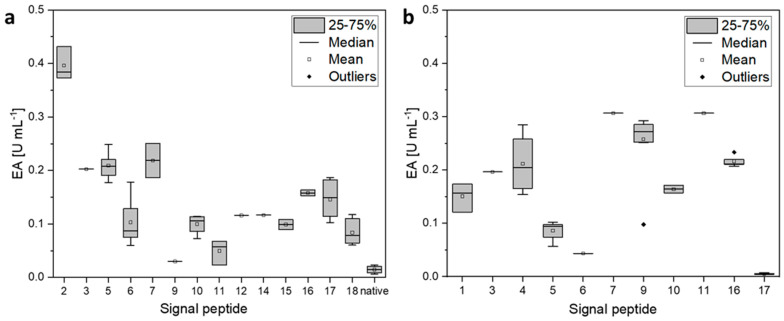
Box plot representation of enzyme activities of BmasPGA in the supernatant obtained by secretion with specific SPs of pMSP3 (**a**) and pMSP4 (**b**). In (**a**), the activity of BmasPGA secreted with the native SPs of all screenings is also plotted.

Next, it was analyzed whether the same SPs in different clones of the screening resulted in similar activity in the supernatant. As only a few clones of SPs of pMSP5 showed activity, the analysis was omitted. It was found that the same SPs resulted in similar activities in the supernatant with only a few outliers such as one clone with SP 9 from pMSP4. This may have resulted from a poorly grown culture, as all other 10 clones with this SP showed a narrow distribution of activity (Figure 7). In summary, all SPs except SP 17 from pMSP4 resulted in higher activity in the supernatant compared with secretion with the native SP (Figure 6 and Figure 7, Table 2).

### 3.6. Scale-Up of Penicillin G Acylase Secretion for Verification of the Screening Results

**Figure 8:**
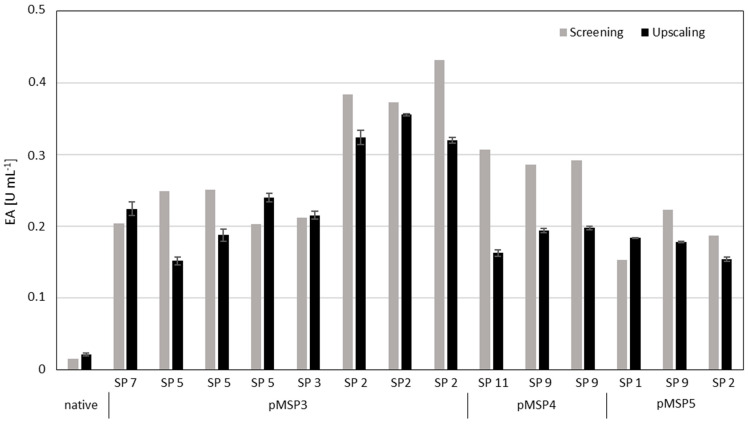
Comparison of volumetric enzyme activity (EA) of selected BmasPGA clones in screening (gray, Figure 6) and upscaling (black). The clones with the native SP as control and the new SPs were cultured at 37 °C in shake flasks (50 mL culture volume). Twenty-two hours after induction of recombinant PGA secretion, the PGA activity was determined in cell-free supernatant by NIPAB assay in triplicates at 37 °C. pMSP3 to 5 are indicating the corresponding multi-SP plasmids; SP X indicates the corresponding SP of the given plasmid as presented in Table 2.

To verify these promising screening results, the best BmasPGA secreting strains were cultivated at 50 mL scale in shake flasks and the cell-free supernatant was analyzed 22 h after induction by measuring enzyme activity and SDS-PAGE analyses of secreted proteins (Figure 8 and Figure S1). All investigated clones showed PGA activities in the supernatant which were between 7.1- and 16.5-fold higher compared to the reference strain with the native SP (Figure 8, Table 3). Additionally, all identified SPs led to a large amount of secreted BmasPGA visible as 25 kDa α-subunit and 60 kDa β-subunit, while for the native SP hardly any secretion was detectable (Figure S1). The highest protein amounts also correlated to the highest measured PGA activities (Figure 8 and Figure S1). Both in screening as well as upscaling, the activity and amount of secreted BmasPGA was the highest with the clones containing the SP of β-amylase (pMSP3-SP 2). Thus, although the increase was slightly smaller than in the screening, the improved secretion based on the alternative SPs could be reproduced on a larger scale. The highest activity of heterologous BmasPGA found (0.36 U mL^−1^) was in the range of native BmPGA (0.5 U mL^−1^) [30] in the cell-free supernatant.

When considering the original proteins guided by the SPs enhancing secretion of BmasPGA, five belong to the enzyme class of hydrolases like the PGA, one belongs to the transferases, three are hypothetical proteins, and one protein is utilized in the biosynthesis of peptidoglycan (Table 3). As the majority of well-suited SPs originate from proteins belonging to the same enzyme class of hydrolases as the PGA, there could be a correlation. A comparison with the secretion of AmyE, also a hydrolase, shows that the use of some SPs such as from the ErfK/YbiS/YcfS/YnhG family protein showed high secretion of AmyE as well as BmasPGA, whereas other SPs, such as from levansucrase, also a saccharide degrading enzyme like AmyE, or conserved hypothetical protein BMD_3012 were among the best SPs for AmyE secretion but under average for BmasPGA secretion.

## 4. Discussion

Due to the lack of the outer membrane, Gram-positive bacteria are well suited to the secretion of recombinant proteins directly to the surrounding growth medium, simplifying protein purification [4,5]. N-terminal SPs mediate the translocation across the cytoplasmic membrane, hence different numbers of SPs have been bioinformatically predicted and identified for a range of Gram-positive bacteria. As early as 2000, Tjalsma et al. predicted 180 potential SPs for *B. subtilis* strain 168, of which 14 were hypothetically assigned to the Tat-dependent and 166 to the Sec-dependent pathway with lengths between 19 and 44 amino acids [38]. Later, 173 of these identified SPs were attributed to the Sec-dependent pathway and tested according to their secretion of different target proteins [17,19]. Additionally, 220 SPs from *Bacillus licheniformis* DSM 13 together with the 173 SPs of *B. subtilis* were applied to optimize the secretion of a protease in both organisms [45]. For *Lactobacillus plantarum* WCFS1, 76 SPs with lengths ranging from 24 to 57 amino acid residues were predicted and applied to improved secretion of a nuclease [21]. For *Corynebacterium glutamicum*, 405 SP candidates were predicted, of which 90 SPs with lengths between 21 to 53 amino acids were proven to be Sec-dependent [46]. In our study, 182 Sec-dependent SPs from *P. megaterium* DSM 319 were predicted, which is in the scope of *Bacillus* species SPs. The lengths of the identified *P. megaterium* SPs range from ten to 56 amino acids. In 2012, Payne and coworkers described the minimal length of SPs for different species to be 10 amino acids separated in two, five, and three residues for n-, h,- and c-domains of the SP, respectively [13]. Hence, although the minimal length of ten amino acids is shorter than the minimal lengths of the SPs described above, these short SPs were included in our screenings to test their functionality. However, most of these short SPs did not show high secretion capacity of our model enzyme α-amylase (Figure 4) so that they were not further analyzed for the following screening of BmasPGA.

The identification and screening of SP libraries for many industrially relevant production hosts mentioned above underlines the importance of improving secretion of recombinant proteins. For this purpose, *P. megaterium* shows lower protease activity in the surrounding medium compared to *B. subtilis* [31] and the introduced recombinant plasmids show high stability [24]. In addition, *P. megaterium* is particularly advantageous because it secretes a remarkably low amount of potentially contaminating host proteins to the surrounding medium so that downstream processing of a recombinant target protein is additionally favored [30] (Figure S1).

So, with the identified *P. megaterium* SPs, a method to improve protein secretion for *P. megaterium* by SP screening using a plasmid-based SP library was established for the first time. Although the prediction of SPs using different tools has been possible for many years [14,15,16], so far it has not been possible to predict the efficiency of a SP for a given protein [19,47] resulting in laborious screening approaches [5]. In previous screenings with all *B. subtilis* SPs, each of the 173 SPs was amplified individually by PCR and *B. subtilis* was transformed individually with the constructed plasmids. This great effort for individual amplification, cloning, and transformation ensured that all SPs were tested [17]. Another possibility is the individual amplification of all 173 *B. subtilis* SPs, and their ligation as a mix into vectors followed by the transformation of *B. subtilis* with the ligation mix [19]. However, the major advantage of our new plasmid-based SP test system is a greatly reduced workload due to the amplification of the coding sequences of the SPs in *E. coli* instead of a PCR amplification before ligation to the target gene. As *P. megaterium* has a low transformation efficiency [39], the transformation of *E. coli* with the ligation mixture was necessary to achieve a high concentration of the ligation products. This step could provide a helpful strategy also for other hosts with low transformation rates such as undomesticated isolates of *B. subtilis* [48], *Lactobacillus plantarum,* or *Lactobacillus buchneri* [49]. For analyzing a small number of specific SPs of *P. megaterium,* individual PCR amplification can be easily used [28]. Nevertheless, the division of SPs into size-related groups of 18 to 20 SPs, each group on one pMSP, reduces the screening effort by organization into individual screening experiments.

The sequencing of SP-coding sequences of our clones from the plasmid-based SPs test system revealed that the individual SPs were represented with different frequencies, which seems to depend on the given recombinant protein (our unpublished data). Fu and coworkers (2018) screened more than 1500 clones for α-amylase secretion based on the 173 SPs of *B. subtilis*, which corresponded to a more than 8-fold oversampling. The SPs of the best 100 clones were sequenced and only 15 different SPs were identified, resulting in up to an 1.7-fold increase in secretion compared to the native SP [19]. In our study, the screening of 86 colonies of a cloning round with SPs from one pMSP represented 78% (14 SPs) of all possible 18 SPs, resulting in a more than 16.5-fold increase of BmasPGA secretion, while even the screening of only 12 clones already represents around 40% of the 18 SPs of another pMSP with an 8.5-fold increase. To cover more or even all SPs, the number of clones to be screened has to be increased accordingly. Anyway, as shown here, testing lower numbers of SPs can be sufficient to identify a suitable SP for secretion.

During the screening and sequencing of SPs applied for α-amylase secretion, it was observed that a high d score, a measure for identification of a SP, does not correlate to strong secretion and vice versa (Table 1) as described previously [17]. This was also observed for the SPs leading to highly enhanced secretion of BmasPGA. Their d scores were found to be only slightly above the threshold value of 0.45, ranging to high values of 0.88 (Table 3). Interestingly, the native SP of BmasPGA also shows a high d score of 0.77 although it hardly leads to recombinant secretion by *P. megaterium*. Consequently, d scores seem to be useful in predicting SPs but cannot valuate them, mainly because the secretion efficiency is determined by the combination of protein and SP and additionally depends on the host organism [5,15]. This could also be shown for the *B. subtilis* SP library, which was applied successfully in *C. glutamicum*, whereas the secretion efficiency of a recombinant protein based on a given SP differed significantly in both organisms [47].

## 5. Conclusions

Our plasmid-based SP library used here is the first SP screening-system for the Gram-positive production host *P. megaterium*. Ours, as well as previous results, confirm that it is still not possible to predict optimally suited SPs for the secretion of recombinant target proteins [19,47]. Therefore, these best SPs need to be identified using a rapid and simple cloning and screening system. In our work, several suited SPs were identified for the secretion of a novel penicillin G acylase from *B. massiliogorillae* (BmasPGA) that resulted in up to a 29-fold increase in the amount of protein secreted compared to secretion via the native SP. With this drastic increase of the required production and secretion to high amounts, the purification from the cell-free supernatant, and the following characterization of the BmasPGA but also of other recombinant novel proteins using *P. megaterium* is possible. Hence, our plasmid-based, easily amplifiable SP library will further widen the application of the interesting production host *P. megaterium* for the secretion of recombinant proteins and additionally could be applied for other hosts due to the functionality of our plasmids in a broad range of *Bacillus* species.

## Figures and Tables

**Table 1:** Signal peptides achieving a better secretion of AmyE compared to the SP of the lipase A (100%).

Signal Peptide	Native Protein ^1^	Secreted AmyE [%] ^2^	d Score	Length
SP 10 from pMSP3	Conserved hypothetical protein BMD_3012 (ADF39852.1)	160.9	0.802	30
SP 6 from pMSP8	Flagellar biosynthetic protein FliZ (ADF40996.1)	160.7	0.660	24
SP 18 from pMSP7	Spore cortex-lytic enzyme (ADF39204.1)	147.0	0.684	24
SP 6 from pMSP8	Flagellar biosynthetic protein FliZ (ADF40996.1)	145.1	0.660	24
SP 9 from pMSP4	ErfK/YbiS/YcfS/YnhG family protein (ADF37131.1)	128.2	0.815	28
SP 12 from pMSP8	Conserved hypothetical protein BMD_0825 (ADF37687.1)	125.5	0.663	23
SP 14 from pMSP3	Levanase (ADF38396.1)	124.0	0.517	29
SP 14 from pMSP3	Levanase (ADF38396.1)	119.3	0.517	29
SP 10 from pMSP3	Conserved hypothetical protein BMD_3012 (ADF39852.1)	117.4	0.802	30
SP 3 from pMSP4	Polysaccharide deacetylase (ADF40719.1)	116.7	0.482	29
SP 6 from pMSP4	Levansucrase (ADF38395.1)	114.7	0.563	29
SP 7 from pMSP4	Phospholipase/carboxylesterase family protein (ADF37374.1)	109.9	0.574	28
SP 10 from pMSP3	Conserved hypothetical protein BMD_3012 (ADF39852.1)	106.8	0.802	30
SP 10 from pMSP3	Conserved hypothetical protein BMD_3012 (ADF39852.1)	106.0	0.802	30
SP 3 from pMSP10	Conserved hypothetical protein BMD_1725 (ADF38580.1)	104.9	0.694	21
SP 10 from pMSP3	Conserved hypothetical protein BMD_3012 (ADF39852.1)	104.2	0.802	30
SP 9 from pMSP9	Conserved hypothetical protein BMD_1305 (ADF38166.1)	102.9	0.562	22
SP 17 from pMSP2	Conserved hypothetical protein BMD_2947 (ADF39787.1)	102.0	0.517	31

^1^ Genebank IDs given in brackets. ^2^ Amount of secreted amylase AmyE [%] compared to the positive control.

**Table 2:** Identity of SPs of pMSP3–5 that led to secretion of BmasPGA.

Signal Peptide	Native Protein	Number of Clones	d Score	Length
**pMSP3**				
none		7		
SP 2	β-amylase	3	0.648	31
SP 3	D-alanyl-D-alanine carboxypeptidase	1	0.875	30
SP 5	Extracellular ribonuclease	4	0.740	30
SP 6	Conserved hypothetical protein BMD_0560	29	0.526	30
SP 7	Alkaline phosphatase	2	0.462	30
SP 9	Putative peptidoglycan binding domain protein	1	0.806	30
SP 10	Conserved hypothetical protein BMD_3012	4	0.802	30
SP 11	Hypothetical protein BMD_3152	3	0.474	30
SP 12	Putative cell wall endopeptidase	1	0.745	30
SP 14	Levanase	1	0.517	29
SP 15	Hypothetical protein BMD_2027	3	0.472	29
SP 16	Bacillolysin precursor (neutral protease)	2	0.888	29
SP 17	VanW family protein	7	0.454	29
SP 18	N-acetylmuramoyl-L-alanine amidase CwlB	14	0.826	29
multiple		4		
**pMSP4**				
SP 1	Hypothetical protein BMD_3427	3	0.451	29
SP 3	Polysaccharide deacetylase	1	0.482	29
SP 4	Signal peptide peptidase SppA, 36K type	4	0.452	29
SP 5	Peptidase M23 family protein	8	0.454	29
SP 6	Levansucrase	1	0.563	29
SP 7	Phospholipase/carboxylesterase family protein	1	0.574	28
SP 9	ErfK/YbiS/YcfS/YnhG family protein	11	0.815	28
SP 10	Conserved hypothetical protein BMD_0309	2	0.660	28
SP 11	Conserved hypothetical protein BMD_0470	1	0.641	28
SP 16	AhpC/TSA family protein	5	0.567	28
SP 17	Spore cortex-lytic enzyme	7	0.835	28
multiple		2		
**pMSP5**				
none		1		
SP 1	Hypothetical protein BMD_2542	1	0.665	28
SP 2	Conserved hypothetical protein BMD_3362	1	0.881	27
SP 9	L-Asparaginase II	1	0.667	27
SP 10	Conserved hypothetical protein BMD_3946	2	0.476	27
SP 11	D-alanyl-D-alanine carboxypeptidase	1	0.758	27
SP 15	Conserved hypothetical protein BMD_1139	4	0.582	26
SP 17	Conserved hypothetical protein BMD_0369	1	0.479	26

**Table 3:** Signal peptides achieving a better secretion of BmasPGA compared to the native SP.

Signal Peptide	Native Protein	Secreted BmasPGA [-] Referred to Native SP ^1^	d Score	Length
native	BmasPGA	1.00	0.772	26
SP 7 from pMSP3	Alkaline phosphatase	10.43	0.462	30
SP 5 from pMSP3	Extracellular ribonuclease	7.06/8.73/11.18	0.740	30
SP 3 from pMSP3	D-alanyl-D-alanine carboxypeptidase	10.03	0.875	30
SP 2 from pMSP3	β-amylase	15.06/16.55/14.90	0.648	31
SP 11 from pMSP4	Conserved hypothetical protein BMD_0470	7.57	0.641	28
SP 9 from pMSP4	ErfK/YbiS/YcfS/YnhG family protein	9.03/9.21	0.815	28
SP 1 from pMSP5	Hypothetical Protein BMD_2542	8.54	0.665	28
SP 9 from pMSP5	L-Asparaginase II	8.28	0.667	27
SP 2 from pMSP5	Conserved hypothetical protein BMD_3362	7.17	0.881	27

^1^ X-fold amount of secreted BmasPGA compared to the native SP.

## Data Availability

The data presented in this study are available within the article.

## Supplementary Materials

The following supporting information can be downloaded at: https://www.mdpi.com/article/10.3390/microorganisms10040777/s1, Table S1: List of plasmids, Table S2: List of primers, Table S3: List of signal peptides available at pMSP1, Table S4: List of signal peptides available at pMSP2, Table S5: List of signal peptides available at pMSP3, Table S6: List of signal peptides available at pMSP4, Table S7: List of signal peptides available at pMSP5, Table S8: List of signal peptides available at pMSP6, Table S9: List of signal peptides available at pMSP7, Table S10: List of signal peptides available at pMSP8, Table S11: List of signal peptides available at pMSP9, Table S12: List of signal peptides available at pMSP10, Figure S1: Comparison of secreted amount of BmasPGA of selected clones in upscaling. Reference [50] is cited in the supplementary materials.
