# Supplementary material for: Construction and Application of a Plasmid-Based Signal Peptide Library for Improved Secretion of Recombinant Proteins with Priestia megaterium
Source: Microorganisms. 2022 Apr 5;10(4):777. doi: 10.3390/microorganisms10040777 (PMC9032162; doi:10.3390/microorganisms10040777)
Supplement: Supplementary file 1 [file microorganisms-10-00777-s001.zip › Mayer et al. Supplementary Material.pdf]

Supplementary Material

Table S1: List of plasmids.

Plasmid	Description	Origin
p3STOP1623hp	Shuttle vector for cloning in *E. coli* (Carb^R) and recombinant protein production in *P. megaterium* (Tet^R) using an optimized xylose-inducible promoter; $P_{xylA^{opt}}$-*mcs-stop*	[1]
pTKSP0	p3STOP1623hp-derivative; shuttle vector for cloning in *E. coli* (Carb^R) and recombinant protein production in *P. megaterium* (Tet^R) with an optimized xylose-inducible promoter, an extended multiple cloning site and His$_6$-tag; $P_{xylA^{opt}}$-*emcs-his$_6$-stop*	This work
pTKSP*amy*E0	pTKSP0-derivative harboring the *amyE* gene from *B. subtilis* 168 without signal peptide inserted via NgoMIV and AgeI	This work
pEJBmD1.3scFv	Shuttle vector for the xylose-inducible recombinant production and secretion of the antibody fragment D1.3 scFv guided by the signal peptide of the lipase A (SP_{LipA}) from *P. megaterium* ATCC 9885 (CAD23620.1); P_{xylA}-*sp$_{lipA}$-d1.3scFv*	[2]
pTKSP*amyE*lipA	pTKSP*amy*E0-derivative containing the signal peptide from lipase A (SP_{LipA}) from *P. megaterium* ATCC 9885 (CAD23620.1) amplified via PCR from pEJBmD1.3scFv [2] and inserted via the restriction sites BsrGI and NgoMIV	This work
pALBm1	p3STOP1623hp-derivative for recombinant production and secretion of PGA from *P. megaterium* (BmPGA); $P_{xylA^{opt}}$-*bmpga-stop*	[3]
pRBBm311	p3STOP1623hp-derivative for recombinant production and secretion of PGA from *Bacillus thermotolerans* (BtPGA); $P_{xylA^{opt}}$-*btpga-stop*	[4]
pRBBm316	p3STOP1623hp-derivative for recombinant production and secretion of PGA from *Bacillus sp. FJAT-27231* (FJATPGA); $P_{xylA^{opt}}$-*fjatpga-stop*	[4]
pRBBm317	p3STOP1623hp-derivative for recombinant production and secretion of PGA from *Bacillus massiliogorillae* (BmasPGA); $P_{xylA^{opt}}$-*bmaspga-stop*	[4]
pJMBm75	pTKSP0-derivative harboring the *bmaspga* gene from pRBBm317 without signal peptide inserted via NgoMIV and NheI	This work
pMSP1 - pMSP9	pUC57-derivative encoding 18 signal peptides of *P. megaterium* DSM 319 separated by the restriction sites BsrGI and NgoMIV, Carb^R, *ori* pBR322	This work, General Biosystems
pMSP10	pUC57-derivative encoding 20 signal peptides of *P. megaterium* DSM 319 separated by the restriction sites BsrGI and NgoMIV, Carb^R, *ori* pBR322	This work, General Biosystems

Table S2: List of oligos.

**Name of oligo**	**Description**	**Sequence**
TKMCSHis_fw	Oligo for insertion of MCS extension, coding sequence of His6-tag and stop codon into p3STOP1623hp; the restriction sites are depicted in italics, the AgeI overhang in bold, the His6-tag in gray and the stop codon in capital letters	**ccgg***cgccg*t*acgcgt*a*cctagg*a*gcggccgcg ctagc*a*accggt*catcaccatcaccatcacTA At
TKMCSHis_rv	Oligo for insertion of MCS extension, coding sequence of His6-tag and stop codon into p3STOP1623hp; the restriction sites are depicted in italics, the AgeI overhang in bold, the His6-tag in gray and the stop codon in capital letters	**ccgg**aTTAgtgatggtgatggtgatg*accg gt*t*gctagcgcggccgc*t*cctagg*t*acgcgt*acg gcg
TKAmyE_fw	Primer for amplification and insertion of *B. subtilis amyE* gene in pTKSP0 resulting in pTKSPamyE0, the NgoMIV site is shown in italics	atcata*gccggc*gaaacggcgaacaaatcg
TKAmyE_rv	Primer for amplification and insertion of *B. subtilis amyE* gene in pTKSP0 resulting in pTKSPamyE0, the AgeI site is shown in italics	atcata*accggt*tcaatggggaagagaacc
TK83fw_LipA_SP	Primer for amplification of lipase A-SP from pEJBmD1.3scFv [2] and insertion into pTKSP*amyE*0 yielding pTKSP*amyE*lipA, the BsrGI restriction site is depicted in italics	atca*tgtaca*atgaaaaaagtattaatggctttc
TK84rev_LipA_SP	Primer for amplification of lipase A-SP from pEJBmD1.3scFv [2] and insertion into pTKSP*amyE*0 yielding pTKSP*amyE*lipA, the NgoMIV restriction site is depicted in italics	atcata*gccggc*ggcgctagcacctgatg
JMSPBmas_fw	Primer for amplification and insertion of bmas*pga* gene from pRRBm317 into pTKSP0 resulting in pJMBm75, the NgoMIV restriction site is depicted in italics	tgatct*gccggc*caaaataacagcggagtgaa ag
JMSPBmas_rv	Primer for amplification and insertion of bmas*pga* gene from pRRBm317 into pTKSP0 resulting in pJMBm75, the NheI restriction site is depicted in italics	tgatct*gctag*cttattttttttcgttttctttgttaa atg
SeqpXylA_fw	Primer for colony PCR and sequencing	aagttggtgtttttttgaagc
SeqpMM1520_rv	Primer for colony PCR	gtttgcgcattcacagttctcc

Table S3: List of signal peptides available at pMSP1

Signal peptide	GenBank ID	Native protein	Sequence
SP 1	ADF38846.1	pullulanase, type I	MITLSKKVIVKNVYVFYFMGGFSLKCSW TKYAMVLTALLLLVSSFFSPFMQSFASA
SP 2	ADF41409.1	protein of unknown function (DUF1510)	MNNNRSSRHENKEKKVNRLYNLLIAVV AVLIVFVGGTMILGNKDKSDNQA
SP 3	ADF37872.1	conserved hypothetical protein BMD_1011	MYRKKTLLLKRSLGVAVFLSVIALGVW MFQFTFFSPSHQAKS
SP 4	ADF37126.1	SH3 domain protein	MEGKQLKRIMVGMALTATLATAVTPGF GSIGGNQEKAYA
SP 5	ADF38520.1	transporter, MmpL family	MKESPLATFSRLMTGKVSRWVVIAVWIL ATAFLTIAWP
SP 6	ADF40203.1	beta-lactamase penicillinase	MKTWKKMFRASKLQKMLPVFALSCAA VVGFSSSEVHA
SP 7	ADF39316.1	alpha amylase	MQTIAKKGDETMKGKKWTALALTLPL AASLSTGVHA
SP 8	ADF37367.1	conserved hypothetical protein BMD_0481	MKTITKSISVLALAAGFTFSSLSGTLPFTH EQTASA
SP 9	ADF38797.1	polysaccharide deacetylase	MNQRFKPNKRFYAFLSLVLVIVASLFFIT EAKGSES
SP 10	ADF39301.1	methyl-accepting chemotaxis protein	MERVRKKRSLNNFKIKLGLAFAAILLVP SVTIGVSA
SP 11	ADF40620.1	conserved hypothetical protein BMD_3787	MKMKQRLSKAAAAATITGIAAFGFAPV AQFDTPAHA
SP 12	ADF41198.1	protein of unknown function (DUF1002)	MGLKEEKGLKLTIKAIMVTLGLLLFLTA PLKSFADA
SP 13	ADF41350.1	cytochrome c-550	MKRNPLIPFLLIAVIGIALMLTLSFNGLN NAKEIAA
SP 14	ADF37697.1	two-component sensor histidine kinase DctS	MRLSMQTKMTGLIFFIVAFSLFLASIVVI NNFVQS
SP 15	ADF40228.1	hypothetical protein BMD_3388	MDFFKNSKILYTIVALVIALIAVFTNNSPI SLIQQ
SP 16	ADF41000.1	flagellar basal body-associated protein FliL	MFKNKLISKMIIIMLSLTLIGSIAVIVTLKY TEQA
SP 17	ADF42034.1	putative peptidase	MIGKVLAGKAVVAKAVGGATIGTAIAL GSMTSAFA
SP 18	ADF38992.1	hypothetical protein BMD_2139	MNISFKKFRFLWFILLIFVLIFVKNTFFSSS QNA

Table S4: List of signal peptides available at pMSP2

Signal peptide	GenBank ID	Native protein	Sequence
SP 1	ADF38011.1	membrane-bound protein, putative transcription regulator	MSSKAKKEWIGGLVLFLLVSFVSI TFAGYHTVKA
SP 2	ADF39126.1	immune inhibitor A metalloprotease	MKSWKKILGTSFLATTVTLSGVG PVLMNGSTASA
SP 3	ADF39213.1	putative membrane protein	MKFIVKFKWVIAAFILALTVILM LTSPNLTKLA

SP 4	ADF39764.1	conserved hypothetical protein BMD_2923	MKLGNQKLIYFSPIIVVAVIFIFILTLIPSASS
SP 5	ADF37892.1	hypothetical protein BMD_1031	MDRKKVASVAGALVIAVGIWLFQSGKFKSEAQA
SP 6	ADF38238.1	conserved hypothetical protein BMD_1377	MKKLIIVAVAAVLLIIGGISFSIYHSSVKDVQS
SP 7	ADF39097.1	conserved hypothetical protein BMD_2249	MVKKLLAAFIALAVVFSPVGGSLIHDGSNVASA
SP 8	ADF39532.1	2',3'-cyclic-nucleotide 2'-phosphodiesterase	MKKNTRSKTKKILSTSLALSLLSMPLLTTQAGA
SP 9	ADF38212.1	cytochrome aa3 oxidase assembly protein	MRLGLKFLSVVTTMAMLLILLGGALVTKTGSA
SP 10	ADF39833.1	Endopeptidase	MKKEKLLVPFIAASVLVGASVTTPVFSHSAYA
SP 11	ADF40159.1	putative hydrolase	MKKVKAALPLAVSLALGLQVLPISTPIVQAEA
SP 12	ADF41059.1	phosphopantothenoylcysteine decarboxylase/phosphopantothenate-cysteine ligase	MMKGKRILLCVSGGIAVYKAAALTSKLVQAGA
SP 13	ADF41166.1	spore cortex-lytic enzyme	MLSFRCFKKYACLICICSFIAASVVCPKAAHA
SP 14	ADF40390.1	putative protease, NlpC/P60 family	MLKKANRTVMNMGLAALLVPAALTFSTTSASA
SP 15	ADF37926.1	endopeptidase LytE	MKKAIIGSITAAAVLFSGASPFIAPAQTVEA
SP 16	ADF38355.1	alkaline phosphatase	MKKRAIGAAVLSAVTVAALAFGTNSPLDAKA
SP 17	ADF39787.1	conserved hypothetical protein BMD_2947	MKKAIVLTYCILSLVLLVSGGLYFKEQMVEA
SP 18	ADF40655.1	polysaccharide deacetylase	MKKYVFFLLIAAALVGFIFFKVVNWEDKAKA

Table S5: List of signal peptides available at pMSP3

**Signal peptide**	**GenBank ID**	**Native protein**	**Sequence**
SP 1	ADF37265.1	conserved hypothetical protein BMD_0354	MMKPIVLNKKIGSIVLIFSLLYFPIAAAAEA
SP 2	ADF37268.1	Beta-amylase	MKQLCKKGLAFVLMFIFVNAFILSPINGAAA
SP 3	ADF36976.1	D-alanyl-D-alanine carboxypeptidase	MRNAVQKQLICVIAVMLMASVFFVSTQASA
SP 4	ADF39725.1	hypothetical protein BMD_2884	MSLSAKKILLLVLVVICLVLVVMQFINRSA
SP 5	ADF37267.1	Extracellular ribonuclease	MKLYALCKKTWAISLLVFVLLVSLTPVSQA
SP 6	ADF37446.1	conserved hypothetical protein BMD_0560	MGDLPLNQMMILVVLLMLSAFFSSAETAFS
SP 7	ADF38066.1	alkaline phosphatase	MRKKLAGITLAGAVALASFGVGNSLIQTEA

SP 8	ADF38542.1	cytochrome C oxidase subunit II, periplasmic domain protein	MSVKNWLMGLVVLFTMVISVM TSGSLGVLA
SP 9	ADF38755.1	putative peptidoglycan binding domain protein	MLKKKTLVTLSTLTLATTCVAFS SPTSEAA
SP 10	ADF39852.1	conserved hypothetical protein BMD_3012	MKSYRLVSFSTLCICLLVLFISPFQ DTSSA
SP 11	ADF39992.1	hypothetical protein BMD_3152	MKMKVRTLFLVLFFAGILVVTSY VDNNSQQ
SP 12	ADF40714.1	putative cell wall endopeptidase	MIWGGNEMKKMLSALLVGGAL FASPAMGHA
SP 13	ADF41182.1	peptidase, M23/M37 family	MIDVLRRILVVLVMGLCIGLLFL GGQMVKA
SP 14	ADF38396.1	levanase	MKKKGAYKWITLAVLLVIIAGLII TAVSK
SP 15	ADF38880.1	hypothetical protein BMD_2027	MEKRHKSKKKRMIISISLSVFLVG GGATG
SP 16	ADF39133.1	Bacillolysin precursor (neutral protease)	MKKKKQALKVLLSVGILSSSFAF AHTSSA
SP 17	ADF39282.1	VanW family protein	MKKVLGALLLGATIVSAFIAYSM YTNHSA
SP 18	ADF39902.1	N-acetylmuramoyl-L-alanine amidase cwlB (Cell wall hydrolase) (Autolysin)	MNKKHVMYTAVSSVLLSSSLLL HSIDADA

Table S6: List of signal peptides available at pMSP4

**Signal peptide**	**GenBank ID**	**Native protein**	**Sequence**
SP 1	ADF40267.1	hypothetical protein BMD 3427	MKWYKHALISLAIGLLSFVCFVI YSKVAA
SP 2	ADF40379.1	conserved hypothetical protein BMD_3539	MKRFKRVSAVVLGLALLFSPLAP QTVAEA
SP 3	ADF40719.1	polysaccharide deacetylase	MKTMGYKRILTLLGCCILFLGTA GYIVKA
SP 4	ADF41608.1	signal peptide peptidase SppA, 36K type	MNKKRWIALLIAACLFIFSAIVNI GFSVK
SP 5	ADF41874.1	peptidase M23 family protein	MTRKTLVMTATILVGLSAAWIG NEKIAYA
SP 6	ADF38395.1	levansucrase	MKMKRVAKHTTAATLAAALLV GGGYQTFA
SP 7	ADF37374.1	phospholipase/carboxylesterase family protein	MYQRLKKSVLLAITLSLAIGIVLS YEKA
SP 8	ADF37703.1	conserved hypothetical protein BMD_0841	MAANKWTKWMVGLSSVAAFTT FIGISQK
SP 9	ADF37131.1	ErfK/YbiS/YcfS/YnhG family protein	MRKIILQAAALFLAAIISISYTASA ANA
SP 10	ADF37220.1	conserved hypothetical protein BMD_0309	MKKKLFGYIMASGLTLGVMAA ANSTAFA
SP 11	ADF37360.1	conserved hypothetical protein BMD_0470	MNIKRWLLTATTVILATFGVSAV SSVHA

SP 12	ADF37807.1	hypothetical protein BMD_0946	MATKRTFFSFFLISALLCLLVVPNSVMA
SP 13	ADF38126.1	spore cortex-lytic enzyme	MKHSYMKSMVVACLATLSFIGFNATATA
SP 14	ADF38532.1	bacterial SH3 domain protein	MKKRIKMLISIGIAASLIIISSQSKVHA
SP 15	ADF39018.1	protease	MKKVSIRSVLSTVAFSVALSSFAMGASA
SP 16	ADF39390.1	AhpC/TSA family protein	MVKKIIAVLLLVGFLSYALWQGLSPNGA
SP 17	ADF40060.1	spore cortex-lytic enzyme	MSKKRVKKWAVASAASVAFLASQGTASA
SP 18	ADF38774.1	hypothetical protein BMD_1921	MKKVLVGMALAFTLVAGVASVGTSVATG

Table S7: List of signal peptides available at pMSP5

Signal peptide	GenBank ID	Native protein	Sequence
SP 1	ADF39387.1	hypothetical protein BMD_2542	MTKRMITLLSVIFIAVLAVFFMSQTSLA
SP 2	ADF40202.1	conserved hypothetical protein BMD_3362	MKKIFPIVLSFGLLTSVSFSTASNVDA
SP 3	ADF40323.1	conserved hypothetical protein BMD_3483	MKKVFNIGVLFFLIFGLFLGVGPTGKA
SP 4	ADF37209.1	subtilisin like serine protease	MKKSLSALLGVSLLVFGLTLGAESSYA
SP 5	ADF38432.1	levanase	MKRTVKKLLMIGVACSISLFSACPVLA
SP 6	ADF39385.1	conserved hypothetical protein BMD_2540	MKKHVKRKVVRILVSLLVVLIIVGSYQ
SP 7	ADF39492.1	conserved hypothetical protein BMD_2648	MEMKKFGIILASLALFFSLSTGVTAQA
SP 8	ADF40338.1	zinc carboxypeptidase family protein	MRKTVKRTAVPALLSIGLLLSPLAVQA
SP 9	ADF40566.1	L-asparaginase II	MKLKRIVSITFLSSVILFSGPLSLVNA
SP 10	ADF40777.1	conserved hypothetical protein BMD_3946	MNRLFKWSASTLLCLMYCFLIPLPSYA
SP 11	ADF41191.1	D-alanyl-D-alanine carboxypeptidase	MKRSKKSIIYLFIVLLCLSGWPSISSA
SP 12	ADF41282.1	stage III sporulation protein AE	MKRWTFKVNILALLLFLFFFFPENVQA
SP 13	ADF41455.1	bypass-of-forespore protein C	MKKVIHQAAFLMLVSIVLCIMPYRADA
SP 14	ADF42031.1	conserved hypothetical protein BMD_5232	MIKWAKVLSTSALGTAIACSSITTSHA
SP 15	ADF38000.1	conserved hypothetical protein BMD_1139	MKAMVGVNVAAAILCGAVMYAGTQHW

SP 16	ADF41919.1	endopeptidase LytF	MKKRQLALSLGAIVGGTFLYQ ASAEA
SP 17	ADF37280.1	conserved hypothetical protein BMD_0369	MKKRFRIATVAGAVSLGLLTA GQANA
SP 18	ADF37788.1	conserved hypothetical protein BMD_0927	MKIFKRMTLAAVTGIFFMTGI TETHA

Table S8: List of signal peptides available at pMSP6

Signal peptide	GenBank ID	Native protein	Sequence
SP 1	ADF37802.1	two-component sensor histidine kinase/response regulator	MNNKLKRKSISTQFILLASIL LLAFA
SP 2	ADF37928.1	modifier protein of major autolysin LytC	MRKLTALALVLSVLLFQFTP LASVKA
SP 3	ADF38462.1	cyanophycinase domain protein	MKKKLVSCLISFTLLSSLSTP LLAKA
SP 4	ADF38750.1	hypothetical protein BMD_1897	MLKIRFALSSLLLVFSLVMG APFASA
SP 5	ADF39236.1	conserved hypothetical protein BMD_2389	MKKFLAMIFVLIFSLSFTFYS STASA
SP 6	ADF39288.1	cell wall-associated protease	MKKKGIISLLSVSLLVSSLSV STTQA
SP 7	ADF39306.1	secreted cell wall DL-endopeptidase	MKKKMLLLNTTVLLGLGGI LSTPAYA
SP 8	ADF39980.1	Lipase/Acylhydrolase (GDSL)	MSSTKKVVFISSIILNVLFLG VFAFY
SP 9	ADF39988.1	transcription regulator domain protein	MKKYKIGILAACILLLGTAG FGIAYQ
SP 10	ADF40077.1	cell wall-associated protease	MNKKGIISLVSAGLLASTLS VQSVQA
SP 11	ADF40397.1	conserved hypothetical protein BMD_3564	MKKFFKIGCLGFIILIALIVVI AVVA
SP 12	ADF40722.1	hypothetical protein BMD_3889	MMKKKLAVISTACLLILASD PIPSYA
SP 13	ADF40824.1	conserved hypothetical protein BMD_3993	MNRVKIIAASLLAVLLLAFS DNFTSA
SP 14	ADF40826.1	glycerophosphoryl diester phosphodiesterase	MRRKWWFVSSLTLCLCGIF MFIHTQA
SP 15	ADF41380.1	conserved hypothetical protein BMD_4556	MIKFTLKSVVLTIFLLCIVFL GMQTA
SP 16	ADF41930.1	integral membrane protein (DUF1779)	MFKKISVFLAIFITGLFVYHG SYVSN
SP 17	ADF40613.1	conserved hypothetical protein BMD_3780	MRKKWSVLLCTLALAMMT FILPVNA
SP 18	ADF37876.1	conserved hypothetical protein BMD_1015	MYIKKCIGSILFLLLFCSSALP AKA

Table S9: List of signal peptides available at pMSP7

Signal peptide	GenBank ID	Native protein	Sequence
SP 1	ADF38398.1	transporter, major facilitator family	MRKRIMTALLLVTVLAAMEGTIVST
SP 2	ADF38764.1	hypothetical protein BMD_1911	MKRKMIALTFSAALCFSASGSLAEA
SP 3	ADF38861.1	foldase protein PrsA	MSKNKIIMIIAAVVIIAAVAIGAVA
SP 4	ADF39025.1	integral membrane protein (DUF6)	MNTSLSKILILCAAVLWGTTGTAQA
SP 5	ADF39788.1	conserved hypothetical protein BMD_2948	MMKLSKKLMIPVLGASLLMPTIANA
SP 6	ADF40634.1	cell wall hydrolase	MKVMKPIMVTVLGLSLAAFYEGAFP
SP 7	ADF41279.1	stage III sporulation protein AH	MLLKKQTVWLLTMLSLVVVLSVYYM
SP 8	ADF37364.1	putative exported cell wall-binding protein	MKKFVFTLGTTAILSAGFVGAASA
SP 9	ADF40809.1	Endopeptidase	MKKVIAALTLAGVIVSTSPIVSQA
SP 10	ADF41873.1	C-terminal processing peptidase	MNRKIVALLMALSLVVGAAGTYFG
SP 11	ADF37363.1	poly(3-hydroxybutyrate) depolymerase	MKRLFIAGIIFILFLSLGAVSSSA
SP 12	ADF37632.1	phosphotransferase system (PTS) lichenan-specific enzyme IIB component	MNILLCCAAGMSTSLLVTKMEKSA
SP 13	ADF37780.1	conserved hypothetical protein BMD_0919	MLGILLIVVAVILVVLIITMNAKA
SP 14	ADF38785.1	polysaccharide deacetylase	MIRRISILLFTLCFLFNGALPALC
SP 15	ADF38838.1	hypothetical protein BMD_1985	MRKFYLAFLLMISTTMVILPSVKA
SP 16	ADF38954.1	spore cortex-lytic enzyme	MFKKLAIIGALAVPMFGMSHGANA
SP 17	ADF39086.1	peptidoglycan-binding protein	MKKHIFTLGATALVSIGIADAASA
SP 18	ADF39204.1	spore cortex-lytic enzyme	MIKKLVIAIVMLIPSFGAGYSAFA

Table S10: List of signal peptides available at pMSP8

Signal peptide	GenBank ID	Native protein	Sequence
SP 1	ADF39501.1	glycerophosphodiester phosphodiesterase	MKKYLAGLSAVALLCCSIASSAFA
SP 2	ADF39554.1	conserved hypothetical protein BMD_2710	MKKTMITFSLVLMSLFGVASGASA
SP 3	ADF39834.1	LysM domain protein	MKKFIFTLGTTAILSAGFVGAASA
SP 4	ADF40014.1	LPXTG-motif cell wall anchor domain protein	MKKLGAILLSFLLFVGVFPTSSFA
SP 5	ADF40015.1	sortase family protein	MEPRKTRRTINRKKRLIVLSLLAA
SP 6	ADF40996.1	flagellar biosynthetic protein FliZ	MLRKFVIAALLISVLAGSNQIVYA
SP 7	ADF41210.1	D-alanine-D-alanine carboxypetidase	MKRTIVKLLVVIFLFPLMTQPVLA
SP 8	ADF41368.1	putative membrane protein	MKTLFSFVCFLILATGLFPTISTA
SP 9	ADF39879.1	cell wall endopeptidase	MKKLIYSLALCGSLTVVPTISEA
SP 10	ADF37299.1	delta-lactam-biosynthetic de-N-acetylase	MRRFTFFLVFLFVMSTGIASVEA

SP 11	ADF37407.1	conserved hypothetical protein BMD_0521	MKKIGLVLSSIAIFFTLSTGVTA
SP 12	ADF37687.1	conserved hypothetical protein BMD_0825	MKKILAFILVIGGTAVLFSTAFA
SP 13	ADF38481.1	endopeptidase LytF (cell wall hydrolase)	MKKIISSLALCSSLIVLPTISQA
SP 14	ADF38842.1	Aminopeptidase	MKKTVISLAVATSLTLSFTSVQA
SP 15	ADF39052.1	conserved hypothetical protein BMD_2204	MTKLMILVFMLISFFVHPQKMKA
SP 16	ADF39606.1	hypothetical protein BMD_2765	MVGFILLFPLNMLAGPISSIAEA
SP 17	ADF39900.1	conserved hypothetical protein BMD_3060	MKRLFVMVAVIAFLALSRMLVFA
SP 18	ADF39990.1	conserved hypothetical protein BMD_3150	MIRKWVAVCVVFAGFIFPQAVSA

Table S11: List of signal peptides available at pMSP9

**Signal peptide**	**GenBank ID**	**Native protein**	**Sequence**
SP 1	ADF40218.1	domain of unknown function (DUF477)	MKFLASFALLLSLLCNMVSVAFA
SP 2	ADF40725.1	gamma-glutamyltransferase	MKVMKMLVIALFSLSLVVTPAAA
SP 3	ADF40822.1	conserved hypothetical protein BMD_3991	MKKTLKIVGVGLAVLVTVTVAFF
SP 4	ADF41772.1	L-Ala--D-Glu endopeptidase	MRKTIILLFMFMSIFTGWHHVTA
SP 5	ADF42035.1	conserved hypothetical protein BMD_5236	MKKRVSLGLSLTLLASSAASAFA
SP 6	ADF39900.1	conserved hypothetical protein BMD_3060	MKRLFVMVAVIAFLALSRMLVFA
SP 7	ADF37524.1	alpha amylase	MKWKRTSMLLILLLLFGSSASA
SP 8	ADF37686.1	conserved hypothetical protein BMD_0824	MKLALLLVIIIGVAALAGTISA
SP 9	ADF38166.1	conserved hypothetical protein BMD_1305	MKKICLLVFLLIVLYSGKSVHA
SP 10	ADF38222.1	putative sporulation protein YpjB	MKGRIVGILLVLFFAYTTTVHA
SP 11	ADF38236.1	conserved hypothetical protein BMD_1375	MIKILISAVLFFSFMNCSVSAA
SP 12	ADF38239.1	aspartate aminotransferase	MKLAKRVSALTPSSTLAITAKA
SP 13	ADF39160.1	conserved hypothetical protein BMD_2312	MRKIMISAAAAFLFMSATPVLA
SP 14	ADF39756.1	polysaccharide deacetylase	MRKISIFVILVLTVIFPSPAFS
SP 15	ADF40784.1	hypothetical protein BMD_3953	MKRYMLSAFLGSLLLLLSIAFS
SP 16	ADF40037.1	hypothetical protein BMD_3197	MKKATSILLIVLALAGLGGVCA
SP 17	ADF37869.1	capsule biosynthesis protein	MNKKLLLFNIIAFLLLICVIA
SP 18	ADF40825.1	conserved hypothetical protein BMD_3994	MKWIGGVFTFSFILFMSSWFA

Table S12: List of signal peptides available at pMSP10

Signal peptide	GenBank ID	Native protein	Sequence
SP 1	ADF40830.1	conserved hypothetical protein BMD_3999	MVKKIALCLLFLMLFPMQINA
SP 2	ADF37228.1	conserved hypothetical protein BMD_0317	MKVITTVLASAVLVGGLGASA
SP 3	ADF38580.1	conserved hypothetical protein BMD_1725	MKKAVGIILILLCCINQRVEA
SP 4	ADF39850.1	Copper resistance protein	MKKIIGLLLACFLAVPTVSFA
SP 5	ADF40036.1	hypothetical protein BMD_3196	MRKWSFLFFVLAFMHPASALA
SP 6	ADF40101.1	competence-specific nuclease	MKKIVSGILILVLAVIVMIEG
SP 7	ADF37283.1	Low molecular weight phosphotyrosine protein phosphatase	MINVLFVCLGNICRSPMAEA
SP 8	ADF40489.1	malate dehydrogenase, NAD-dependent	MAFRRKKIAVIGAGFTGATA
SP 9	ADF40755.1	hypothetical protein BMD_3923	MKRMRAFAIMTFLVVGSGYA
SP 10	ADF40786.1	conserved hypothetical protein BMD_3955	MKRLFYTVSTFCFVWLAFA
SP 11	ADF37472.1	conserved hypothetical protein BMD_0586	MSRMKKLAAGAAIGSTVAA
SP 12	ADF37968.1	hypothetical protein BMD_1107	MLKKIAVLLMMLGIAHF
SP 13	ADF38163.1	Ser/Thr protein phosphatase family protein	MKKTYTRRSFLKG
SP 14	ADF39761.1	two-component sensor histidine kinase PhoR	MKISLKAKMGLV
SP 15	ADF41187.1	cytochrome c biogenesis protein	MKKKRLIIRTVI
SP 16	ADF41329.1	penicillin-binding protein	MKKKKRRKSHV
SP 17	ADF41793.1	conserved hypothetical protein BMD_4993	MSKLKKDPSSA
SP 18	ADF37949.1	diguanylate cyclase/phosphodiesterase	MSIKKKLSVF
SP 19	ADF39130.1	conserved hypothetical protein BMD_2282	MRKRKQKALI
SP 20	ADF39284.1	conserved hypothetical protein BMD_2438	MTVRKRKRKK

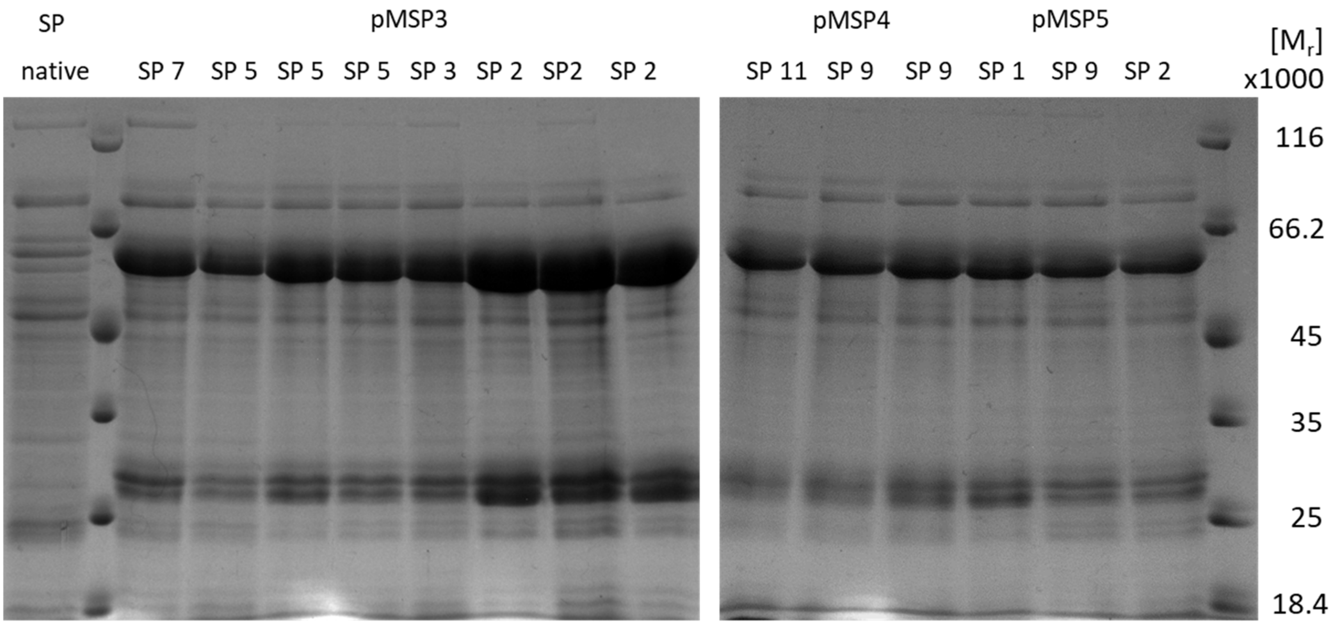

Figure S1: Comparison of extracellular proteins of BmasPGA-secreting selected clones of recombinant *P. megaterium* in upscaling. The clones with the native SP as control or with the alternative SPs were cultured at 37 °C in shake flasks (50 mL culture volume). Twenty-two hours after induction the extracellular proteins were precipitated by ammonium sulfate. pMSP3 to 5 are indicating the corresponding multi SP plasmids, SP X indicates the corresponding SP of the given plasmid as presented in Table 2.

References

1. Stammen, S.; Müller, B.K.; Korneli, C.; Biedendieck, R.; Gamer, M.; Franco-Lara, E.; Jahn, D. High-yield intra- and extracellular protein production using *Bacillus megaterium*. *Appl. Environ. Microbiol.* **2010**, *76*, 4037–4046.

2. Jordan, E.; Hust, M.; Roth, A.; Biedendieck, R.; Schirrmann, T.; Jahn, D.; Dübel, S. Production of recombinant antibody fragments in *Bacillus megaterium*. *Microb. Cell Fact.* **2007**, *6*, 1–11.

3. Lakowitz, A.; Krull, R.; Biedendieck, R. Recombinant production of the antibody fragment D1.3 scFv with different *Bacillus* strains. *Microb. Cell Fact.* **2017**, *16*, 1–18.

4. Mayer, J.; Pippel, J.; Günther, G.; Müller, C.; Lauermann, A.; Knuuti, T.; Blankenfeldt, W.; Jahn, D.; Biedendieck, R. Crystal structures and protein engineering of three different penicillin G acylases from Gram-positive bacteria with different thermostability. *Appl. Microbiol. Biotechnol.* **2019**, *103*, 7537–7552.
